# Myocardial oxygen consumption, myocardial efficiency, and mechanical efficiency: A review across pathologic and physiologic states

**DOI:** 10.14814/phy2.70890

**Published:** 2026-05-21

**Authors:** Julia A. Pierson, Ludmil Mitrev, Jeffrey C. Hill, Lawrence J. Mulligan

**Affiliations:** ^1^ Cooper Medical School of Rowan University Camden New Jersey USA; ^2^ Department of Anesthesiology Cooper University Hospital Camden New Jersey USA; ^3^ School of Medical Imaging and Therapeutics Massachusetts College of Pharmacy and Health Sciences University Worcester Massachusetts USA

**Keywords:** aortic stenosis, heart failure, myocardial efficiency, myocardial oxygen consumption (MVO_2_), noninvasive cardiac assessment

## Abstract

Myocardial work and oxygen consumption (MVO_2_) are tightly coupled. The patient's physiological state, presence of disease, heart rate, and microvascular control of oxygen delivery all combine to maintain this relationship. The myocardial efficiency (MyoEff) of the heart is determined by the ratio of stroke‐work (SW) divided by MVO_2_, whereas mechanical efficiency (ME) is derived from the left ventricular pressure‐volume loop construct and is the ratio of SW to pressure‐volume area. The need to collect invasive data limited the ability to perform human studies, but the development of noninvasive tools such as positron emission tomography (PET) combined with echocardiography led to a resurgence in this field of study. We summarize the current understanding of the significance of MyoEff and ME as outcomes predictors after aortic valve replacement and as parameters in cardiovascular health, highlighting their potential for improving risk stratification and guiding prophylactic interventions.

## 1 INTRODUCTION

**FIGURE 1:**
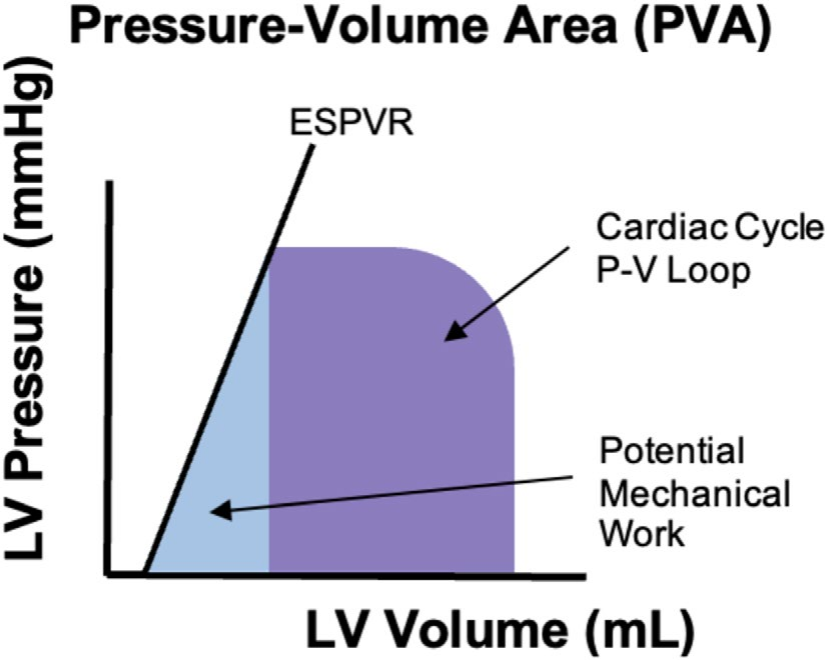
A schematic of the left ventricular pressure‐volume construct. The slope of the relation, ESPVR (end‐systolic pressure‐volume slope) provides a load‐independent measure of the contractile state. The area under the slope enables the quantification of potential energy (PE).

**FIGURE 2:**
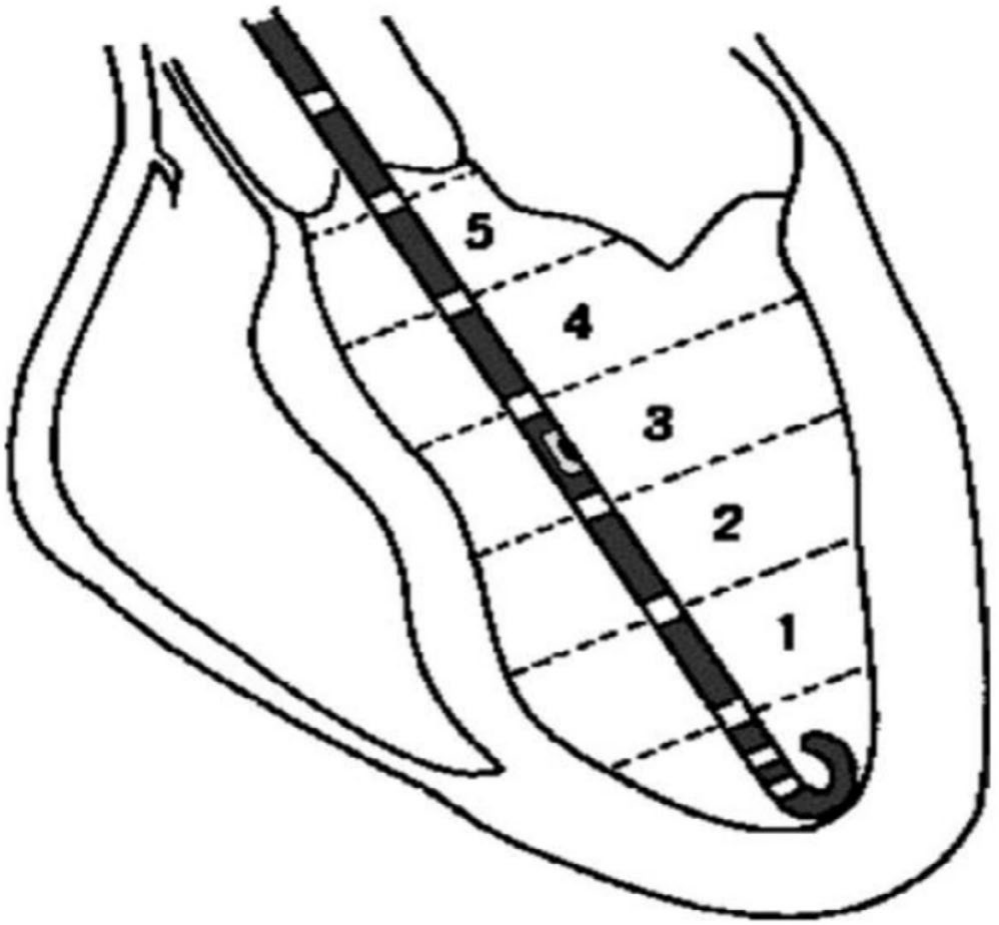
A schematic of the left ventricular pressure‐volume conductance catheter. The regions 1–5 are summed to create the total left ventricular volume and coupled with the left ventricular pressure to create the PV loop in Figure 1.

Cardiovascular disease (CVD) continues to be a significant global health challenge and is growing despite the recognition of modifiable risk factors (Joynt Maddox et al., 2024). To improve primary prevention and guide treatment of common diseases, an improvement in easy‐to‐use noninvasive parameters is needed to provide focused care and enhance risk stratification. Pressure‐volume (PV) analysis is a foundational concept in quantifying ventricular energetics (Figure 1). It shows the relationship between left ventricular pressure and volume throughout one cardiac cycle. Key metrics extracted from the PV loop include stroke volume (SV), ejection fraction (EF), and stroke work (SW). The PV concept allows for calculation of stroke work (SW), which is the area contained in the PV loop, equivalent to external, or useful, work, and the pressure‐volume area (PVA), which represents total mechanical energy expenditure. PVA is linearly correlated with myocardial oxygen consumption, linking the pressure‐volume loop concept with important clinical insights into multiple disease states (Takaoka et al., 1992). This review highlights invasive and noninvasive measures of myocardial oxygen consumption (MVO_2_) and myocardial efficiency (MyoEff), as defined by stroke work (SW)/MVO_2_. MVO_2_ is the amount of oxygen utilized by the cardiomyocytes per unit time to maintain cellular viability and perform cardiac work. It encompasses the basal metabolic needs of cardiomyocytes and the energy required for mechanical contraction. This review also explores mechanical efficiency (ME), a concept derived from the left ventricular pressure‐volume loop construct, equal to the ratio of stroke work to pressure‐volume area (PVA). ME and MyoEff reflect two distinct aspects of cardiac function: the proportion of stroke work performed by the heart compared to PVA, and stroke work as a proportion of oxygen consumption (Table 1). While ME and MyoEff provide a view of the pump efficiency and metabolic efficiency respectively, they are not interchangeable. In recent years, MyoEff has regained attention as an important metric for its use as a treatment target in heart failure (Hansen et al., 2022). The current approach requires either positron emission tomography (PET) or the use of the pressure‐work index (PWI), requiring invasive catheterization (Figure 2). The PWI was developed using the Rooke and Feigl metric, which correlates work to estimated myocardial oxygen consumption, approximated using the rate‐pressure product (RPP) (Rooke & Feigl, 1982). This review will evaluate how measures of ventricular energetics including MVO_2_, MyoEff, and ME can be used to stratify disease states and guide interventions using noninvasive measures of cardiac function.

**TABLE 1:** Ventricular energetics terms and interpretations.

Term	Definition
Stroke work (SW)	Area contained by the pressure‐volume loop, representing external work performed by the heart.
Pressure‐volume area (PVA)	Sum of stroke work and potential energy, representing total mechanical energy expenditure.
Myocardial oxygen consumption (MVO_2_)	Oxygen consumption of cardiomyocytes per unit time determined by heart rate, contractility, and wall stress. Linear relationship to PVA
Myocardial efficiency (MyoEff)	Equal to SW/MVO_2_
Mechanical efficiency (ME)	Equal to SW/PVA
RPP (rate pressure product)	Heart Rate multiplied by Systolic Arterial Pressure
MEE	Mechano‐energetic efficiency
MEEi	MEE indexed for myocardial mass

## 2 FOUNDATIONAL WORK

Mechanical efficiency (ME) and myocardial efficiency (MyoEff) have been investigated in canine models (Freeman & Colston, 1990; Nozawa et al., 1994) and numerous clinical studies (Table 2). The collection of these data requires an invasive study of left ventricular pressure‐volume metrics relying on skilled operators with intensive training to navigate the aortic valve by means of a catheter (Figure 2). Total myocardial energy consumption (MVO_2_) represents a collection of hemodynamic related metrics (e.g., wall tension, contractility, and heart rate) and intracellular biochemical components including enzymes of substrate metabolism, mitochondrial respiratory chain components, and sarcolemma proteins (Myrmel & Korvald, 2000).

**TABLE 2:** Early studies of MyoEff.

Author (year)	Patient state	Sample size	Sex of participants	MyoEff (%)
Bing and Hammond (1949a, 1949b)	Healthy	4	Male	22 ± 2
Baxley et al. (1977)	HF/VHD	38	Male	21 ± 9
Nichols et al. (1986)	Healthy	8	6 Male, 2 Female	29 ± 6
	CAD	15	14 Male, 1 Female	26 ± 10
	CAD + MI	13	Male	16 ± 5^a^
Sundram et al. (1990)	HF	9	Male	10 ± 5
Beanlands et al. (1993)	HF	8	4 Male, 4 Female	13 ± 5

Abbreviation: MyoEff‐SW: myocardial efficiency‐stroke work.

^a^ Compared to healthy (*p* < 0.001).

## 3 CARDIAC METABOLIC BACKGROUND AND CLINICAL IMPLICATIONS

Cardiomyocytes contain the highest volume of mitochondria of any cell in the human body, occupying about one third of the intracellular space (Schaper et al., 1985) making dysregulated metabolism an important factor in many cardiac disease states. Myocardial mitochondrial function is primarily driven by oxidative phosphorylation, supplied by all types of substrates including carbohydrates, lipids, amino acids, and ketone bodies (Taegtmeyer et al., 2016). Although fatty acids are the preferred substrate, providing the highest total ATP yield per molecule, cardiomyocytes are highly flexible “omnivores”, and are able to undergo substrate switching when physiological conditions change. Cardiac mitochondria are under several regulatory control mechanisms including post‐translational modifications and allosteric regulation (Kolwicz Jr. et al., 2013). For example, the heart undergoes dramatic metabolic shifts as the fetal heart transitions from glucose dependence to fatty acid preference mediated by peroxisome proliferator‐activated receptor‐gamma coactivator‐1 (PCG‐1) α and PGC‐1 β (Lai et al., 2008). Adaptation to reduced oxygen availability is driven by hypoxia‐inducible factor 1 (HIF‐1) transcription which protects against reactive oxygen species by increasing the efficiency of complex IV in the electron transport chain and activating the gene that encodes pyruvate dehydrogenase kinase 1 (PDK1) to deactivate pyruvate dehydrogenase (PDH) and reduce pyruvate flux through the mitochondria (Semenza, 2011). PDK4‐mediated phosphorylation of PDH is a critical post‐translational modification; similarly, this requires the heart to rely on fatty acid oxidation for energy production (Schafer et al., 2018). An important allosteric regulator of substrate utilization is malonyl‐CoA, an allosteric inhibitor of carnitine palmitoyltransferase‐I (CPT‐I), which is the rate‐limiting enzyme for fatty acid uptake in the mitochondria. As utilization of glucose increases, malonyl‐CoA levels rise through acetyl‐CoA carboxylase activity, blocking fatty acid entry into mitochondria and encouraging glucose metabolism. Decreased levels of myocardial malonyl‐CoA have been implicated in disease states including heart failure, obesity, and diabetes (Ussher & Lopaschuk, 2008), showcasing the utility of underlying basic science in clinical correlates.

## 4 CLINICAL INVESTIGATIONS: INITIAL STUDIES

Investigations of these variables began more than 75 years ago and involved invasive techniques (Bing & Hammond, 1949a, 1949b). Bing and Hammond provided the initial insights regarding myocardial oxygen consumption (MVO_2_) and myocardial efficiency in humans (Bing & Hammond, 1949a). Baxley et al. (1977) sought to apply their methods to obtain cardiac measurements of patients with heart failure and/or valvular disease. Multiple studies in a variety of clinical states expanded their findings (Table 1). Nichols et al. (1986) evaluated myocardial efficiency across three patient populations: patients with normal coronary angiograms, patients with angiographically revealed coronary artery disease (CAD), and patients with CAD and a prior myocardial infarction (MI). They found that MyoEff declined significantly in those with CAD and MI compared to healthy patients, yet patients with isolated CAD and no history of MI were similar to controls. Sundram et al. (1990) collected invasive measurements of myocardial function in heart failure patients with no coronary artery disease. Using increasing doses of dobutamine and amrinone, they found that there were additive benefits which improved myocardial efficiency without increasing oxygen consumption.

Suga (1990) provides a comprehensive framework linking ventricular energetics to PVA and demonstrates the value of invasive (conductance catheter) and noninvasive (imaging) measurement compared to approximations including time‐tension index or wall stress.

**FIGURE 3:**
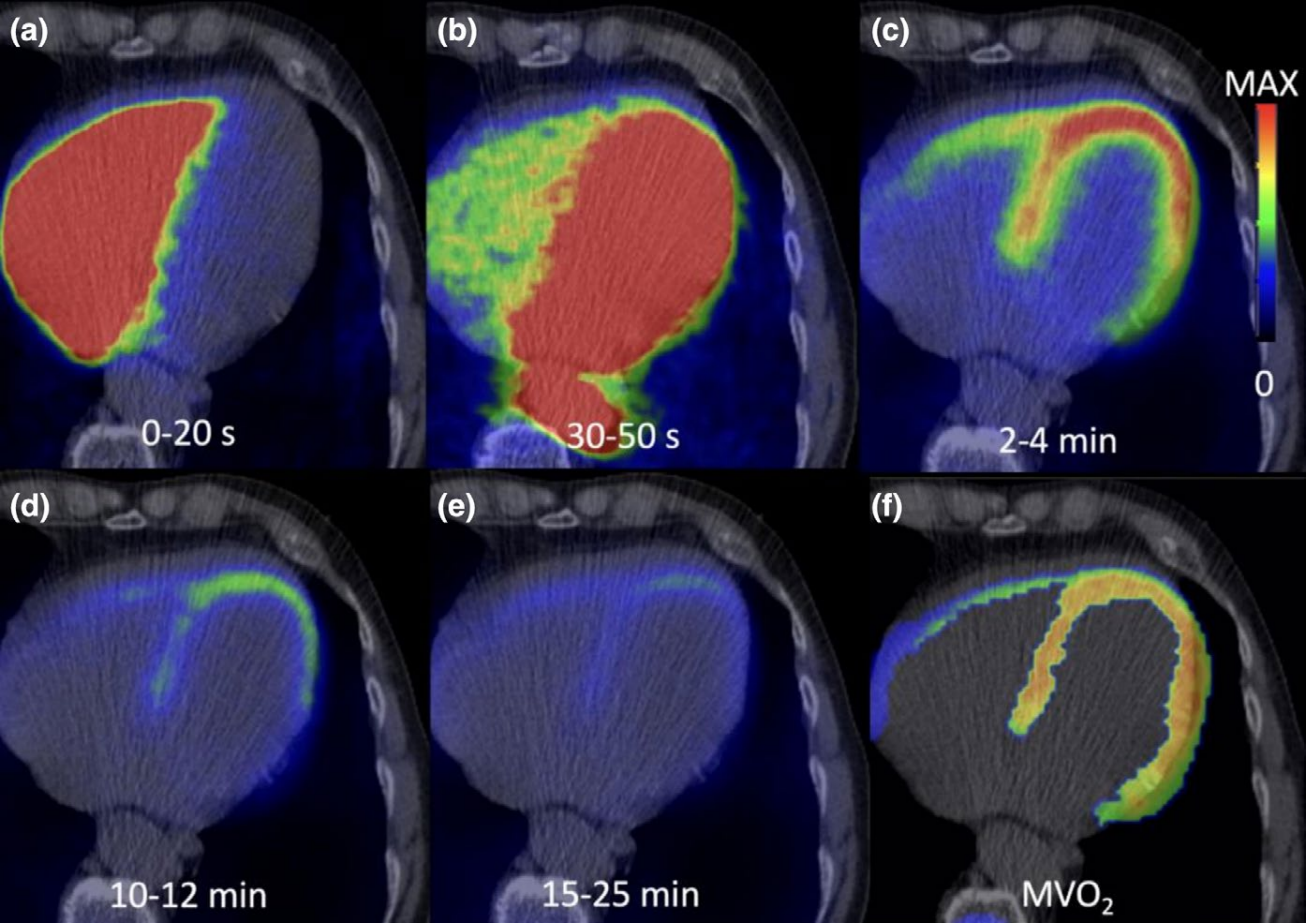
Illustrated example of ^11^C‐acetate PET adopted from Sorensen et al. (2020). (a) initial bolus in the right ventricle, (b) distribution to the left heart, (c) uptake by the myocardium, (d, e) metabolism by the myocardium, (f) clearance rate is assessed for each voxel, plotted as oxygen uptake.

The use of the left ventricular pressure‐volume (PV) construct along with the calculation of MVO_2_ eliminated the necessity of invasive clinical studies. With the development of C‐11 acetate PET, noninvasive studies combining echocardiography and PET provided a method that enables both the calculation of MVO_2_ and MyoEff (Yamamoto et al., 1996). PET is especially useful since a single scan simultaneously quantifies left ventricular mass (LVM), end‐systolic wall stress (ESWS), and myocardial blood flow (MBF) (Sorensen et al., 2020). This method works by using labeled carbon (C‐11) to dynamically calculate the rate of the tricarboxylic acid (TCA) cycle flux which is linearly correlated with MVO_2_ in both animal and human studies (Figure 3) (Sun et al., 1997, 1998; Weiss et al., 1992). Beanlands et al. (1993) evaluated patients presenting with nonischemic heart failure using invasive catheterization to validate PET as a method to measure MyoEff.

Finally, Vanoverschelde et al. (1993) compared PET‐derived MVO_2_ and echocardiographic measures of SW to calculate MyoEff in eight healthy males. Importantly, this study also used the Rooke and Fiegl algorithm providing a simple calculation of estimated MVO_2_ (eMVO_2_) using only blood pressure and echocardiography‐derived stroke volume, heart rate, body weight, and left ventricular mass. Vanoverschelde's study included a baseline collection of the metrics followed by infusion of dobutamine and demonstrated that PET‐derived MVO_2_ and eMVO_2_ were highly correlated (*r* = 0.92); however, this tool did not gain use in clinical studies. Using a computational model to simulate how changes in afterload and heart rate impact the ME and MyoEff metric, Mulligan et al. (2025) found that eMVO_2_ values were concordant with physiologic values of MyoEff across several interventions and in agreement with previous PV loop studies.

## 5 CLINICAL INVESTIGATIONS: PV LOOP STUDIES

After the development of the conductance catheter that provided the pressure‐volume (PV) data along with instrumentation for measurement of MVO_2_, a number of studies were conducted that assessed ME (Kameyama et al., 1992; Takaoka et al., 1993; Vanoverschelde et al., 1993). The use of the left ventricular PV construct along with the calculation of MVO_2_ provided novel insights into several patient populations including heart failure, transcatheter aortic valve implementation, and cardiac resynchronization therapy (Bastos et al., 2020). Yet, the invasive nature of this approach limited MyoEff as a tool for current use. Indirect parameters include the rate‐pressure product (RPP) and the triple rate‐pressure product (TRP: stroke volume × RPP).

## 6 MYOCARDIAL OXYGEN CONSUMPTION AND MYOCARDIAL EFFICIENCY, AND MECHANICAL EFFICIENCY ACROSS PATHOLOGIC AND PHYSIOLOGIC STATES

### 6.1 Physiologic changes after chronic endurance exercise

Exercise‐induced cardiac remodeling (EICR) represents a set of structural and functional changes to the heart. In activities with a high dynamic and low static components, like long distance running, the heart undergoes eccentric LV remodeling. In contrast, activities with low dynamic and high static components, such as weightlifting, the heart undergoes concentric LV hypertrophy. Interestingly, in activities with high dynamic and static components like boxing or rowing, the heart experiences eccentric LV hypertrophy (LVH) characterized by both LV chamber expansion and wall thickening (Baggish et al., 2017). In addition to a thorough clinical history, EICR can be distinguished from pathological states by assessing noninvasive imaging such as echocardiography. These findings include mild, symmetric LVH with walls less than 15 mm and normal valve morphology. Any diastolic dysfunction should raise the suspicion for underlying pathology, as LV diastolic function is increased in athletic hearts. Regular aerobic exercise leads to increased capillary density and surface area which enhances the capacity for oxygen exchange between the blood and myocardial tissue (Koller et al., 2022). Exercise is theorized to provide extensive cardioprotective effects including increased capacity for glycolytic flux, changes in nitric oxide signaling, amplified endoplasmic reticulum stress proteins, improved function of sarcolemmal and mitochondrial ATP‐sensitive potassium channels, and elevated antioxidant capacity (Powers, 2017). Endurance training enhances mechanical efficiency by increasing LV mass and compliance to augment stroke volume and cardiac output. In response to 1 year of intensive endurance training, sedentary individuals' hearts underwent eccentric hypertrophy after 6–9 months of training for a marathon‐like endurance event (Arbab‐Zadeh et al., 2014). Adjusted for gram of myocardium, MVO_2_ is lower in endurance athletes; however, estimated myocardial efficiency is similar between athletes and untrained participants, reflecting the metabolic adaptations attained from long‐term endurance exercise.

### 6.2 Physiologic changes during normal pregnancy

In normal pregnancy, cardiac output increases by 30%–50%, primarily due to increased stroke volume, essential for maintaining adequate perfusion to both the mother and the fetus (Tan & Tan, 2013). The heart undergoes eccentric hypertrophy due to the increased volume load and stroke work. Therefore, left ventricular mass and end‐diastolic volume increase during pregnancy, reflecting the heart's adaptation to the increased workload (Savu et al., 2012). Diastolic function may decrease as pregnancy progresses, indicated by impaired relaxation and increased filling pressures. Yet in comparison to disease states, maternal cardiac metrics typically return to baseline three to 6 months postpartum (Savu et al., 2012). Iacobaeus et al. (2018) explored cardiac function during pregnancy and found that myocardial energetic efficiency was maintained, despite the rapid increase in LV mass, heart rate, and cardiac output (Table 3). This demonstrates that the myocardium can function efficiently in normal pregnancy, including in the third trimester when structural changes and diastolic dysfunction are most prominent.

**TABLE 3:** Assessment of MVO_2_ and myocardial efficiency in aortic stenosis, heart failure, obesity, hypertension, and insulin sensitivity, cardiac amyloidosis, inflammatory arthritis, and pregnancy.

Author, year	Disease state	Technique/method	*N*	Sex of participants	MVO_2_	Efficiency
Güçlü et al. (2015)	Aortic stenosis, pre and post valve replacement	Echocardiography, cardiopulmonary exercise test, [11C]‐acetate PET and cardiovascular magnetic resonance imaging	14 Control	9 Male, 5 Female	Did not assess	Control 49 ± 6%
			10 AVS	7 Male, 3 Female	Pre AVR: 0.11 ± 0.03 mL/min/g	Pre AVR: 32 ± 7%
					Post AVR: 0.09 ± 0.02 mL/min/g (*p* = 0.02 vs. pre)	Post AVR: 37 ± 5% (*p* = 0.02 vs. pre)
Hansson, Sörensen, Harms, Kim, Nielsen, Tolbod, Frøkiær, Bouchelouche, Dodt, Sihm, & Poulsen (2017); Hansson, Sörensen, Harms, Kim, Nielsen, Tolbod, Frøkiær, Bouchelouche, Dodt, Sihm, Poulsen, & Wiggers (2017)	Aortic Stenosis	11C‐Acetate PET, Echocardiography	10 Control	7 Male, 3 Female	0.11 ± 0.02 mL/min/g	21.0 ± 1.6%
			37 Asymptomatic AS LVEF ≥50%	25 Male, 12 Female	0.12 ± 0.02 mL/min/g	22.3 ± 3.3%
			12 Symptomatic AS LVEF ≥50%	7 Male, 5 Female	0.13 ± 0.03 mL/min/g	22.1 ± 4.2%
			9 Symptomatic AS LVEF <50%	7 Male, 2 Female	0.1 ± 0.03 mL/min/g	17.3 ± 4.7% (*p* < 0.05 vs. all)
Cetin et al. (2018)	HF	Echocardiography, mean blood flow using coronary sinus antegrade flow measurements	20 Control	14 Male, 6 Female	1.06 ± 0.22 cal/systole	25.7 ± 12.4%
			80 HFrEF	67 Male, 13 Female	0.78 ± 0.49 cal/systole	13.4 ± 19.0% (*p* = 0.008)
Mancusi et al. (2021)	Obesity /Hypertension (by quartile of MEE)	Echocardiography, blood pressure	111	64 Male, 47 Female	Did not assess	Lowest quartile of MEE < 41%
			120	50 Male, 70 Female		42%–54%
			125	40 Male, 85 Female		54%–67%
			124	31 Male, 93 Female		Highest quartile of MEE >67%
Mancusi et al. (2019)	HOMA‐IR (<1 indicates insulin sensitivity)	Echocardiography, blood pressure	784 (<1.71)	376 Male, 408 Female	Did not assess	0.48 mL*g/s
			776 (1.72–2.75)	326 Male, 450 Female		0.47 mL*g/s
			785 (2.76–4.66)	322 Male, 463 Female		0.46 mL*g/s
			783 (>4.67)	298 Male, 485 Female		0.44 mL*g/s
Clemmensen et al. (2018)	Cardiac amyloidosis	11 C‐acetate PET, Echocardiography, right heart catheterization	10 Control	3 Male, 7 Female	0.10 ± 0.02 mL/min/g	24 ± 5%
			25 CA	20 Male, 5 Female	0.09 ± 0.02 mL/min/g (*p* = 0.22)	13 ± 5% (*p* < 0.0001)
Cioffi et al. (2021)	Inflammatory arthritis	Echocardiography, blood pressure	216 Control	91 Male, 125 Female	Did not assess	0.45 ± 0.10 mL*g/s
			432 Chronic Inflammatory Arthritis	158 Male, 274 Female		0.35 ± 0.11 mL*g/s (*p* < 0.001)
Iacobaeus et al. (2018)	Pregnancy	Echocardiography	31 First Trimester	Female	Did not assess	0.40 mL*g/s
			31 s Trimester			0.35 mL*g/s
			31 Third Trimester			0.37 mL*g/s
			31 Post Partum (9 mo)			0.41 mL*g/s

### 6.3 Heart failure

Heart failure can be stratified into states of reduced ejection fraction (HFrEF) stemming from systolic dysfunction or preserved ejection fraction (HFpEF) which can be associated with diastolic dysfunction. Using transthoracic echocardiography (TEE), Cetin et al. (2018) found that HFrEF patients had lower ME and MyoEff, but MBF was preserved (Table 3). They measured that a decrease in ME by one calorie increased the risk of cardiovascular events 4.3 times, and a decrease in MyoEff by 1% increased the risk 3.3 times, concluding that echocardiography‐derived ME and MyoEff are independent predictors of adverse outcomes in HFrEF. In HFpEF patients, Losi et al. (2019) suggest that impaired myocardial energy balance, reflected by low MyoEff and adjusted for LV mass, plays a significant role in disease progression. While LVH and diastolic dysfunction are commonly associated with HFpEF, a substantial proportion of these patients do not exhibit clear LVH or diastolic dysfunction. Low MyoEff could help identify phenotypes at high risk even in the absence of these traditional markers of HFpEF. The study highlights the importance of energy production and utilization in HFpEF pathophysiology, suggesting that shifting focus from hemodynamics and cardiac mechanics to energy metabolism may be productive.

### 6.4 Hypertension

Arterial hypertension, defined as systolic blood pressure greater than or equal to 130 mmHg or diastolic blood pressure greater than or equal to 80, is implicated in the development of heart failure. In early hypertension, before LVH develops, MVO_2_ per gram of myocardium is increased due to increased stroke work and afterload but normalizes as concentric remodeling occurs, increasing the weight of the myocardium (Laine et al., 1999). Myocardial efficiency is decreased in concentric LVH due to lower stroke volume. Impaired efficiency paired with reduced coronary perfusion reserve leaves the left ventricular wall vulnerable to ischemia, leading to the development of HFrEF. Low mechano‐energetic efficiency adjusted for myocardial mass (MEEi) can detect preclinical LV impairment and progression toward HF in hypertensive patients using PET and computed tomography (CT) (Lembo et al., 2023).

### 6.5 Metabolic syndrome

Metabolic syndrome increases the risk of developing CVD. To fit the diagnostic criteria for metabolic syndrome, patients must be positive for three of five risk factors: abdominal obesity, elevated triglycerides, low HDL, systolic blood pressure over 130 mmHg or diastolic blood pressure over 85 mmHg, fasting glucose over 110 mg/dL (6.11 mmol/L) (Grundy et al., 2005).

Insulin resistance negatively impacts myocardial efficiency by disrupting the heart's metabolic flexibility. Fatty acid oxidation, the normal source of myocardial adenosine triphosphate (ATP), is less energy efficient than glucose oxidation, requiring 30%–50% more oxygen for a given amount of ATP when adjusted for stroke work (Murphy et al., 2016). However, in the healthy population, using glucose as a substrate for ATP production in the myocardium is not favorable due to the limited glycogen available in the myocardium (Lionetti et al., 2011). The ratio of ATP produced to oxygen consumed is higher using glucose than fatty acids as a substrate. In disease states, there is a shift toward increased glucose utilization, since an increase in myocardial oxygen efficiency attempts to compensate for deficits in mechanical work, up to the point of failure (Lionetti et al., 2011). Insulin resistance decreases myocardial efficiency and contributes to CVD independently from hypertension, lipid profile, and central obesity (Mancusi et al., 2021) (Table 3).

In obese individuals without CVD, cardiac metabolism is also characterized by inefficient ATP production and utilization, promoting subclinical systolic dysfunction (Mancusi et al., 2021). Mancusi et al. (2019) were able to demonstrate that in obese individuals, the left ventricle functions with high levels of energy wasting when corrected for LV mass (Table 3). In another study using cardiac magnetic resonance in overweight subjects, increased myocardial triglycerides were associated with increased LV mass and reduced systolic function, factors that are associated with lower MVO_2_ (Szczepaniak et al., 2003). Additionally, increased epicardial adipose tissue can directly create alterations in myocardial structure and function (Neeland et al., 2013).

### 6.6 Aortic stenosis

MVO_2_ is typically increased in aortic stenosis (AS) due to the LV pressure overload and resultant hypertrophy. ME is often reduced in AS as the disease progresses and in the presence of LV dysfunction (Güçlü et al., 2015). MVO_2_ and ME are important aspects of cardiac function in patients with AS that may be involved in the transition from compensated left ventricular hypertrophy to heart failure, as AS represents pressure overload of the left ventricle. In compensated AS, MVO_2_ per gram of myocardium may remain near normal, but total MVO_2_ is elevated due to increased LV mass and wall stress. As AS severity increases, the heart's ability to generate stroke work is impaired, leading to reduced myocardial external efficiency (MEE) (Naya et al., 2010) (Table 3). In the study by Hansson, Sörensen, Harms, Kim, Nielsen, Tolbod, Frøkiær, Bouchelouche, Dodt, Sihm, & Poulsen (2017), utilizing 11C‐acetate PET and echocardiography, MVO_2_ remained consistent across groups with varying stages of AS symptoms (Table 3). However, MEE was significantly reduced in symptomatic patients with left ventricular ejection fraction (LVEF) <50%. MEE was also impaired in patients with low‐flow, low‐gradient AS, reaching similar levels to those with reduced LVEF. The MEE reduction in these groups was primarily driven by an inability to maintain external stroke work rather than changes in total MVO_2_. This suggests that mitochondrial oxidative phosphorylation remains intact even as hypertrophy and heart failure develop. In the study by Bahlmann et al. (2021), low mitochondrial energetic efficiency (MEEi) was associated with significant cardiac and all‐cause mortality adjusted for age, AS, and hypertension. Interestingly, they found reduced survival in these patients with mild to moderate AS compared to severe AS which may represent a high‐risk population of asymptomatic AS patients.

### 6.7 Cardiac amyloidosis

Cardiac amyloidosis (CA) is characterized by the deposition of amyloid in myocardial tissue, leading to myocardial stiffness, diastolic dysfunction, and restrictive cardiomyopathy. The most common forms of CA are light chain (AL) amyloidosis and transthyretin (ATTR) amyloidosis, each named for their respective protein product. The accumulated cardiotoxic proteins disrupt oxidative metabolism by uncoupling mitochondria (Falk et al., 2016). Amyloid deposition can progress to HFpEF and ultimately lead to systolic dysfunction, compromising cardiac output. Cardiac amyloid fibrils disrupt myocardial architecture and function, leading to increased myocardial oxygen consumption with reduced myocardial external efficiency (Clemmensen et al., 2018) (Table 3). In this study, they coupled invasive (Swan‐Ganz catheterization) and noninvasive (11C‐acetate PET) myocardial work parameters and found that the reduction in MEE is associated with decreased systolic performance and increased symptom burden.

### 6.8 Inflammatory arthritis

Patients with chronic inflammatory arthritis, including those with rheumatoid arthritis, ankylosing spondylitis and psoriatic arthritis, often develop associated cardiac disease. Cioffi et al. (2021) used echocardiography to calculate MEE in patients with chronic inflammatory arthritis to stratify their risk of cardiovascular mortality or hospitalization (Table 3). They found that more than one third of patients with inflammatory arthritis had low MEE, independent of confounding variables, and lower MEE was a prognostic indicator for cardiovascular events. The chronic inflammation associated with these conditions is thought to shift energy production toward aerobic glycolytic energy production instead of fatty acid oxidation (Lacourt et al., 2018). Chronic inflammation has also been shown to increase insulin resistance, which was more likely to be found in inflammatory arthritis patients with a low MEE.

### 6.9 Impact of SARS‐CoV‐2 on myocardial energetics

The SARS‐CoV‐2 virus alters MVO_2_ by processes that create oxygen demand–supply mismatch (Giustino et al., 2020). This occurs through four main mechanisms: elevated sympathetic tone and fever increase heart rate and myocardial work, endothelial injury occurs in the coronary microcirculation, increased angiotensin II‐mediated vasoconstriction, and hypoxemia from ARDS or microthrombi impair perfusion. Concurrently, myocardial injury and inflammation caused by elevated IL‐6 and TNF‐α concentrations reduce contractile efficiency, resulting in a higher oxygen cost per unit of stroke work (Chang et al., 2023). These factors create a state of energetic inefficiency which traditional indices such as rate–pressure product tend to underestimate. This is because RPP does not capture metabolic demand and cannot reflect tissue‐level oxygen delivery (Pecchiari et al., 2021). This phenotype parallels septic physiology, characterized by a dissociation between hemodynamic measures and myocardial oxygen utilization, and highlights the need for integrative, noninvasive measures of myocardial efficiency in COVID‐19–associated cardiovascular dysfunction.

### 6.10 Anesthesia and surgical implications

Evidence shows that MEE relates to postoperative functional improvement after valve surgery, but links to broad anesthesia‐specific outcomes (e.g., technique/agent choice) are not well established yet. This provides a rich area for future perioperative research involving MEE and related parameters. Lower pre‐operative MEE in severe AS improves after surgical and transcatheter aortic valve replacement (AVR) and predicts the degree of post‐operative functional recovery (e.g., exercise capacity/LV performance) (Güçlü et al., 2015). Circulatory efficiency in AS is frequently reduced before surgery, and normalizes in most patients after AVR, aligning with the idea that energetic efficiency tracks surgical benefit (Nordmeyer et al., 2021). Further work is needed to establish the exact associations of MyoEff, ME and MEE with noncardiac surgical outcomes, or whether the choice of anesthetic technique/agent (volatile vs. propofol) can serve as a modulator of MEE‐linked perioperative risk independent of the anesthetic effects on ischemia–reperfusion injury and myocardial protection.

## 7 THERAPEUTIC STRATEGIES TO IMPROVE MYOCARDIAL FUNCTION

Cardioactive medications, including guideline‐directed medical therapy (GDMT) used to treat heart failure, often have an impact on ventricular energetics. Interventions targeting metabolic processes include SGLT‐2 inhibitors, metformin, pioglitazone, and GLP‐1 receptor agonists. Although the latter three have been shown to reduce macrovascular and major adverse cardiovascular events (MACE), they do not have a significant impact on cardiac function or myocardial metabolism. As a pillar of GDMT, SGLT‐2 inhibitors such as dapagliflozin and empagliflozin have cardioprotective effects, shifting metabolic utilization from excessive glucose to energy‐efficient substrates like fatty acids, branched‐chain amino acids, and ketone bodies in patients with and without type 2 diabetes (Mudaliar et al., 2016). Beta‐blockers, another pillar of GDMT, reduce myocardial oxygen demand by decreasing heart rate, blood pressure, and contractility to prolong coronary perfusion time. Metoprolol has been shown to reduce MVO_2_ and improve myocardial efficiency in patients with asymptomatic aortic stenosis despite the concern that beta blockade confers negative inotropic effects (Hansson, Sörensen, Harms, Kim, Nielsen, Tolbod, Frøkiær, Bouchelouche, Dodt, Sihm, Poulsen, & Wiggers, 2017).

## 8 CONCLUSION

Myocardial mechanical efficiency and myocardial oxygen efficiency are important parameters in cardiovascular health. From the initial invasive studies by Bing and Hammond to the development of noninvasive PET scans, the assessment of MyoEff and ME has evolved considerably. Studies across diverse clinical conditions demonstrate the utility of these parameters in understanding disease progression and predicting outcomes. By integrating these parameters into clinical practice, physicians can gain a more comprehensive understanding of cardiac function and tailor interventions to improve patient outcomes. While the clinical focus should be tailored to patients' disease states, future research of noninvasive monitoring of MyoEff should aim to provide early warning signs of CVD and enable monitoring of disease progression. Further work is needed to establish the exact associations of MyoEff, ME, and MEE with surgical outcomes, or whether the choice of anesthetic technique/agent (volatile vs. propofol) can modulate MEE‐linked perioperative risk independent of the anesthetic effects on ischemia–reperfusion injury and myocardial protection.

## AUTHOR CONTRIBUTIONS

**Lawrence J. Mulligan:** Conceptualization; methodology. **Ludmil Mitrev:** Writing – review & editing. **Julia A. Pierson:** Methodology. **Jeffrey C. Hill:** Conceptualization.

## FUNDING INFORMATION

The authors have nothing to report.

## ETHICS STATEMENT

None.

## Data Availability

The original contributions presented in the study are included in the article. Further inquiries can be directed to the corresponding author.
